# Adiposity Measures and Plasma Adipokines in Females with Rheumatoid and Osteoarthritis

**DOI:** 10.1155/2017/4302412

**Published:** 2017-11-01

**Authors:** Vanessa DeClercq, Yunsong Cui, Cynthia Forbes, Scott A. Grandy, Melanie Keats, Louise Parker, Ellen Sweeney, Zhijie Michael Yu, Trevor J. B. Dummer

**Affiliations:** ^1^Population Cancer Research Program, Department of Pediatrics, Dalhousie University, Halifax, NS, Canada; ^2^School of Health and Human Performance, Dalhousie University, Halifax, NS, Canada; ^3^Centre of Excellence in Cancer Prevention, School of Population and Public Health, University of British Columbia, Vancouver, BC, Canada

## Abstract

The objective of this study was to examine the relationship between adipokines and adiposity in individuals with rheumatoid and osteoarthritis in the Atlantic PATH cohort. Using a nested case-control analysis, participants in the Atlantic PATH cohort with rheumatoid or osteoarthritis were matched for measures of adiposity with participants without a history of arthritis. Both measured and self-reported data were used to examine disease status, adiposity, and lifestyle factors. Immunoassays were used to measure plasma markers. BMI was positively correlated with percentage body fat, fat mass index (FMI), and a change in BMI from 18 years of age in all 3 groups. There were no statistical differences between levels of plasma adipokines; adiponectin levels were 6.6, 7.9, and 8.2 *μ*g/ml, leptin levels were 10.3, 13.7, and 11.5 ng/ml, and resistin levels were 10.0, 12.1, and 10.8 ng/ml in participants without arthritis, with rheumatoid arthritis, and with osteoarthritis, respectively. Those with higher levels of adiponectin were more likely to have osteoarthritis (but not rheumatoid arthritis). No association was found between arthritis types and leptin or resistin. This study demonstrates differences in measures of adiposity and adipokines in specific types of arthritis and highlights the need for more research targeting specific adipokines during arthritic disease progression.

## 1. Introduction

Arthritis is a condition that affects joints and the surrounding tissues, causing chronic pain, limiting mobility, and contributing to disability. The Atlantic Provinces have some of the highest rates of arthritis in Canada; the overall prevalence of arthritis in Canada is approximately 16%, whereas Newfoundland and Labrador, Nova Scotia, and New Brunswick each report a prevalence of 25% [1].

Research suggests that there are nonmodifiable risk factors such as age, sex, and genetics that impact the risk of developing arthritis. For example, data from the Canadian Community Health Survey in 2014 indicated similar rates of individuals who report being diagnosed with arthritis at younger ages, but at 45 years of age, the prevalence of arthritis is higher in females than in males [1]. Modifiable risk factors including physical activity, diet, and maintaining a healthy body weight may also prevent disease progression, reduce pain, and improve movement [2].

Excess body weight is associated with many comorbidities that decrease lifespan, produce a substantial economic burden, and increase the risk of multiple chronic diseases. Similar to the high rates of arthritis, the percent of individuals that are obese in Atlantic Canada (24–30%) is well above the national average (20%) [3]. Meta-analyses have demonstrated a dose response in the prevalence of both rheumatoid and osteoarthritis with increasing body mass index (BMI) [4–7].

Earlier work suggested that a simple mechanical mechanism was responsible for the relationship between obesity and arthritis. But more recently, associations between obesity and non-weight-bearing joints suggest that the mechanism might be more complex, involving adipose-tissue-derived proteins that have immunomodulatory and bone remodeling properties [8]. In obesity, there are substantial changes in the inflammatory mediators (cytokines and adipokines) secreted from adipose tissue [9], which may regulate physiological actions in tissue and cells of the joints involved in arthritic disease progression [10, 11]. The adipokine known as adiponectin is generally considered anti-inflammatory, and circulating levels decrease with obesity. However, in the case of arthritis, adiponectin may act as a proinflammatory mediator by promoting the degradation of joint tissues [11, 12]. Thus, the complex relationship between these adipokines in arthritis is not yet fully understood and direct comparisons of adipokine levels in both lean and obese individuals with specific types of arthritis (osteoarthritis versus rheumatoid) are lacking. While rheumatoid arthritis has long been viewed as an inflammatory condition, more recently, we have started to understand that inflammatory mediators also play a crucial role in the initiation and progression of osteoarthritis [13, 14]. Therefore, the objective of this study was to investigate the association between plasma adipokine levels in specific types of arthritis (rheumatoid and osteoarthritis) among a cohort of Atlantic Canadians.

## 2. Methods

### 2.1. Study Population and Data Collection

Questionnaire data, physical measures, and biological samples were collected between 2009 and 2015 from participants aged 35 to 69 years that were residents of New Brunswick, Newfoundland and Labrador, Prince Edward Island, and Nova Scotia, Canada. The questionnaires included data on sociodemographic, health, environmental, and lifestyle factors. Recruitment and data collection has been previously described [15]. All individuals provided written informed consent prior to participation. The original data and sample collection were approved by the Research Ethics Boards in each Atlantic Canadian province and the secondary analysis of biological samples was approved by the Dalhousie University Research Ethics Board (#2016-3884).

### 2.2. Assessment of Arthritis, Obesity, and Lifestyle Risk Factors

The prevalence of arthritis among Atlantic Canadians was derived from the self-reported incidence of arthritis in the Atlantic PATH cohort. Participants were asked to respond “yes” or “no” if a doctor had ever told them that they had arthritis. If yes, participants indicated the age at first diagnosis as well as the type of arthritis by checking: rheumatoid arthritis, osteoarthritis, do not know, or other. In the current study, there were 30549 participants that provided data on ever being diagnosed with arthritis, and of those participants, anthropometric measures such as height and weight were provided by 24569 participants. As part of the questionnaire, participants were asked to self-report anthropometric data including height, weight, and waist and hip circumference measurements. Additionally, body composition (i.e., fat mass and fat-free mass) was measured by bioelectrical impedance, using a Tanita Segmental Body Composition Analyzer (Tanita BC-418), and standard anthropometric measures (height, weight, and hip and waist circumference) were collected by research nurses at assessment centres. Both self-reported and measured height, weight, and waist and hip circumference were used for calculating BMI and waist-to-hip ratios. BMI values were used to quantify the percent of Atlantic PATH participants who were underweight, normal weight, overweight, or obese as defined by having a BMI of <18.5, 18.5–24.9, 25.0–29.9, or >30.0, respectively. Additional adiposity measures, waist-to-hip ratio, and waist circumference are also useful to identify individuals at an increased risk of obesity-related comorbidities and were used as measures of body fat distribution in this study. The waist-to-hip ratio was set at >0.85 for women [16] and abdominal obesity was defined as having a waist circumference ≥ 88 cm for women [15, 16]. The fat mass index and fat-free mass index were calculated by dividing fat mass and fat-free mass by height in meters squared, respectively [17]. Questions related to lifestyle and behavior, including diet, physical activity, smoking, alcohol consumption, and education were assessed. Education was categorized as high school or lower, college level, and university level or higher. For smoking behavior, participants were asked if they had smoked at least 100 cigarettes in their life and if they answered no, they were classified as never smoked. If yes, participants further answered the question, at the present time, do you smoke cigarettes daily, occasionally (at least one in the past 30 days), or not at all (did not smoke at all in the past 30 days)? And they were classified as a current smoker (daily or in the past 30 days) or former smoker (has not smoked in the past 30 days). For alcohol consumption, participants were classified as an abstainer, occasional drinker (<3 times/month), regular drinker (1–3 times/week), and habitual drinker (4–7 times/week). For fruit and vegetable intake, total servings of fruit and vegetables consumed in a typical day were used.

### 2.3. Assessment of Physical Activity

Participants were asked to report their levels of physical activity using open-ended questions in the International Physical Activity Questionnaire (IPAQ long form). Participants were asked to identify the frequency and duration of all vigorous, moderate, and walking PA (i.e., inclusive of occupational, domestic, and leisure time) within the last seven days. Each question defined the specific intensity and domain, as well as provided examples. Using the IPAQ guidelines for data processing and analyses, daily and weekly metabolic equivalents of a task (MET) values were calculated for each domain. Total score of PA in MET (minutes/week) were calculated by summing up all MET values across all domains. In accordance with the criteria of IPAQ scoring protocol, categorical PA score was defined as inactive, moderately active, and active.

### 2.4. Biological Samples

In addition to the questionnaire and physical measures data, a subgroup of 21160 participants provided biological samples. Blood samples were collected from participants via arm venipuncture into vacutainer tubes by a phlebotomist. For plasma samples, blood was collected in EDTA-containing tubes, inverted 8 times then stored at 4°C for transport. Samples were shipped on ice to a central processing facility where they were centrifuged at 1500*g* for 10 minutes at 4°C. Samples were processed within 24 hours of collection, then aliquoted into cryotubes and placed in −80°C freezers for long-term storage. Plasma levels of adipokines were measured by magnetic bead-based immunoassays; adiponectin was measured as a singleplex assay (171-A7003M, Bio-Rad Laboratories), whereas leptin and resistin were measured together in a multiplex assay (171-A7001M, Bio-Rad Laboratories). For measurements of adiponectin, samples were diluted 1 : 400, and for detection of leptin/resistin, samples were diluted 1 : 4. Data were acquired on the Bio-Plex MAGPIX and Bio-Plex 200 systems (Bio-Rad). The intra- and inter-assay coefficients of variation were ≤10 and 15%, respectively. Data were analyzed in duplicate using the Bio-Plex Manger 6.0 software (Bio-Rad).

Since adipokine levels are influenced by obesity [9] and participants in the Atlantic PATH cohort with arthritis had higher reported measures of adiposity compared to those without arthritis (data not shown), we conducted a nested case-control study (*n* = 240) of participants with rheumatoid, with osteoarthritis, or without arthritis that were matched for BMI (Table 1). Females were more likely than males to have arthritis, and arthritis was also increasingly prevalent with advancing age (data not shown). Thus, to simplify the analysis and avoid sex/age-based variations, the nested case-controlled analysis included only female Atlantic PATH participants over the age of 50 years who self-identified as being diagnosed with a specific type of arthritis (e.g., rheumatoid or osteoarthritis).

### 2.5. Statistical Analysis

Categorical variables are described as frequencies and percentages, and continuous variables are described as means ± standard deviation. Data analyses were performed with SPSS (Version 22 IBM) and statistical significance was defined as *P* ≤ 0.05. The Kolmogorov-Smirnov test was used to test for normal distribution of data. If normally distributed, groups were compared with ANOVA, and nonnormally distributed groups were compared with the Kruskal-Wallis test. Pearson's correlation coefficients were calculated to assess correlations between different adiposity measures in participants without arthritis, with rheumatoid arthritis, or with osteoarthritis. Multinomial logistic regression models were used to assess adipokine concentrations as predictors of incidence of arthritis.

## 3. Results

### 3.1. Characteristics of Participants

The participants in each of the arthritis groups were older compared to the group without arthritis. Lifestyle risk factors such as smoking, alcohol consumption, education, fruit and vegetable intake, and physical activity level were similar between all three participant groups (Table 1). Participants in the case-controlled analysis not only were matched for BMI, but also had similar measures of waist circumference, waist-to-hip ratio, percent body fat, FMI, BMI at 18 years, and a change in BMI from 18 years of age (Table 1).

Since the correlation between BMI and fat mass varies with age, sex, and disease condition such as rheumatoid cachexia [18–20], we examined the correlation between measures of adiposity in each of the study groups. There was a significant correlation between several of the adiposity measures in participants without arthritis, with rheumatoid arthritis, and with osteoarthritis (Table 2). More specifically, BMI was positively correlated with percentage body fat, FMI, and change in BMI from 18 years of age. No significant association between BMI, waist circumference, or waist-to-hip ratio was found in any of the groups (Table 2). FMI was positively correlated with BMI, percent body fat, and a change in BMI from 18 years of age (Table 2) in all groups. Waist circumference was positively correlated with the waist-to-hip ratio in all groups. Waist-to-hip ratio was inversely correlated with BMI, percentage body fat, and FMI in participants with rheumatoid arthritis (Table 2).

### 3.2. Plasma Adipocytokine Levels

**Figure 1:**
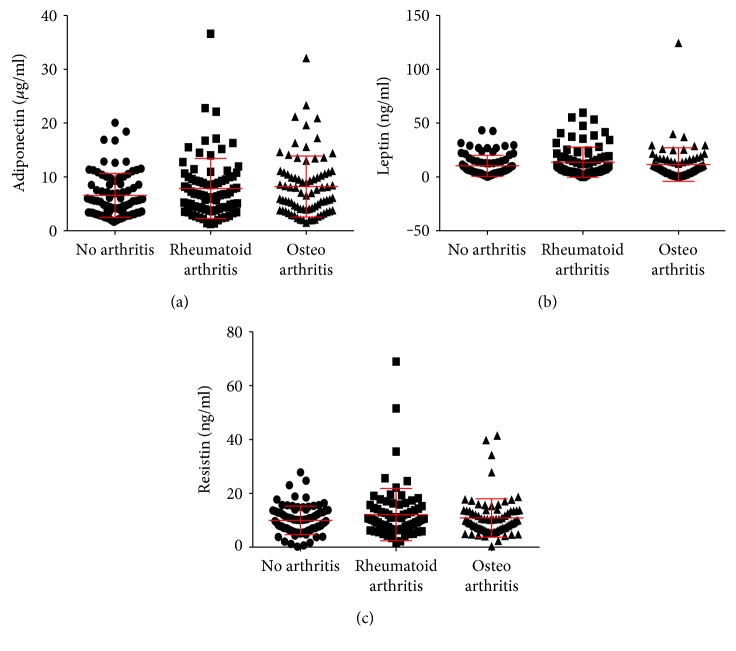
Plasma (a) adiponectin, (b) leptin, and (c) resistin in participants without arthritis, with rheumatoid arthritis, and with osteoarthritis. Horizontal lines represent means ± standard deviations, and dots, squares, and triangles represent individual data points from participants without arthritis, with rheumatoid arthritis, and with osteoarthritis, respectively. Data are not statistically different as determined by the Kruskal-Wallis test.

Plasma adiponectin levels were 7.9, 8.2, and 6.6 *μ*g/ml in participants with rheumatoid, with osteoarthritis, and without arthritis, respectively (*P* = 0.16, *n* = 240) (Figure 1(a)). Plasma leptin levels were 13.7, 11.5, and 10.3 ng/ml in participants with rheumatoid arthritis, with osteoarthritis, and without arthritis, respectively (*P* = 0.34, *n* = 240) (Figure 1(b)). Plasma resistin levels were 12.1, 10.8, and 10.0 ng/ml in participants with rheumatoid arthritis, with osteoarthritis, and without arthritis, respectively (*P* = 0.49, *n* = 240) (Figure 1(c)).

Logistic regression analysis found no significant association between adipokines and rheumatoid arthritis (Table 3). In contrast, plasma adiponectin levels were significantly associated with osteoarthritis (Table 4). This relationship remained after adjusting for age, education, smoking, alcohol use, and fruit and vegetable consumption (Table 4). There was no association between plasma leptin or resistin levels and osteoarthritis (Table 4).

## 4. Discussion

Arthritis is among the most commonly reported chronic conditions in Canada and although associations with BMI have been reported, the underlying molecular mechanisms are not well understood. This study demonstrates a significant association between several measures of adiposity (BMI, percent body fat, and FMI) in female participants with arthritis and shows that of several adipokines assessed, only adiponectin was associated with a specific type of arthritis.

Previous studies have reported that only 24% of women with arthritis have a BMI ≥ 30.0 [6], whereas a higher prevalence of participants with arthritis (38%) had a BMI in the obese range in the Atlantic PATH cohort (data not shown). Although, with the ever increasing obese population [3] and increased incidence of arthritis in those over the age of 45–55 years [21], the prevalence in the current cohort may not be all that surprising. Several others have reported a higher incidence of arthritis in those with increasing BMI [4, 7, 22, 23]. However, BMI is not the only measure associated with excess body weight. Waist circumference, waist-to-hip ratio, and percent body fat/fat mass are also commonly associated with the metabolic complications of obesity [24–26]. Some studies have attempted to include more than one variable [27–30]; however, there is a lack of research directly comparing these distinct measures of adiposity in individuals with different types of arthritis. The results of the current study show a strong association between different measures of adiposity such as BMI, percent body fat, FMI, and a change in BMI from 18 years of age in participants without arthritis and those with rheumatoid arthritis or osteoarthritis. BMI at 18 years of age has been shown to be a strong predictor of obesity in adulthood, and gain in BMI has been associated with increased risk of disease (e.g., diabetes and hypertension, osteoarthritis, and rheumatoid arthritis) [31–33].

Similar to our results, a significant association has been reported between BMI and percent fat mass, as well as the correlation between percent fat mass and waist-to-hip ratio [28]. Likewise, percent body fat and FMI were reported to be significantly higher in patients with rheumatoid arthritis compared to controls [34]. Previous research suggests that BMI and percent body fat are associated with osteoarthritis of the hand, knee, and hip in both sexes [28–30]. In addition, an association with the waist-to-hip ratio in patients with hand osteoarthritis has been documented [28]. We did not observe a significant association in waist-to-hip ratio and other adiposity measures in participants with either form of arthritis or without arthritis; however, we were not able to differentiate between the location of arthritis in the current study. Adiposity measurements in relationship to specific locations of arthritic joints may be considered in future research.

The relationship linking arthritis and obesity is complex and additional investigation is needed to better understand the complex pathophysiology of the disease. Obesity is a state of chronic low-grade inflammation and it is possible that the deterioration and loss of cartilage in arthritis may be due to an imbalance between proinflammatory and anti-inflammatory cytokines that result in catabolic effects on cartilage [9–11, 35]. Recent reports suggest that factors secreted directly from the adipose tissue, adipokines, may be involved in the inflammatory process, as well as cartilage and bone remodeling [8]. Thus, we examined the relationship between systemic markers (such as adipokines) and arthritis in obese individuals with specific types of arthritis.

Previous research into the molecular causes of rheumatoid arthritis has shown mixed results with some studies reporting no difference or increased levels of adiponectin in those with rheumatoid arthritis compared to those without [36]. Although we observed slightly higher levels of both adiponectin and leptin in participants with rheumatoid arthritis, no significant association was found. In contrast, a meta-analysis of 6 prospective cohort studies showed that leptin levels were higher in rheumatoid patients with higher disease activity [37]. Interestingly, the duration of rheumatoid arthritis ranged from 14 months to 17 years and leptin levels were highest in those with the shortest duration of disease. Although we did not measure disease activity in the current study, the average duration of rheumatoid arthritis among participants was almost 16 years. Yoshino et al. examined the same 3 adipokines as the current study and reported both higher levels of leptin and adiponectin in females with rheumatoid arthritis but similar levels of resistin [38]. Importantly, the abovementioned study reported differences of adipokines levels between sexes and was examining a population with an average BMI within the normal range. These distinctions between participants and studies may be important when considering what types of adipokines may be potential therapeutic targets in the future.

Much of the previous research in osteoarthritis suggested higher circulating adipokine levels in osteoarthritis patients [39–44], and although we observed higher adiponectin and leptin levels in participants with osteoarthritis, only adiponectin resulted in a significant association with osteoarthritis. It is worth noting that the extent of osteoarthritis may influence adipokine levels as associations with adipokines and grading or severity of disease have been documented [12, 40, 42]. One study which examined adiponectin, leptin, and resistin found that all 3 adipokines were elevated in those with osteoarthritis; however, these patients all had severe osteoarthritis and were significantly older than control participants [42]. In addition, the location of osteoarthritis may also be worth considering when reporting inflammatory mediators. For example, higher IL6 were reported in both the synovial fluid and serum of patients with hip osteoarthritis, whereas serum leptin levels have been reported to be higher in patients with knee osteoarthritis [45]. A limitation of the current study is that no data on the location site of arthritis was collected. Perhaps different cytokines and adipokines are distinct at various locations and stages of the disease process. Furthermore, adipokines may be more predictive as markers when combined with other established biomarkers of osteoarthritis or inflammatory mediators involved in cartilage degradation. For instance, adiponectin levels have been shown to correlate with inflammatory mediators as well as enhance the secretion of inflammatory mediators from cultured cartilage [12, 46].

A strength of the current study is that multiple measures of obesity including BMI, waist circumference, waist-to-hip ratio, and body composition were utilized to assess adiposity. In addition, blood samples and objectively measured anthropometric variables on the majority of participants in the Atlantic PATH cohort are also major strengths. As with any self-reported data, there are disadvantages, including issues related to participant recall of disease and other health and lifestyle factors. The Atlantic PATH data was limited to the self-report of the disease history, age of diagnosis, type of arthritis, and there was no site-specific information provided. Site-specific arthritis may be considered for future studies as a high BMI has been associated with an increased odds ratio of osteoarthritis of the knee and hand but not the hip [47]. As well, pharmacological management of arthritis was not taken into account prior to examining the nested case-control sample. Accordingly, future research should explore the use of medications that may influence plasma adipokines levels in these populations. Furthermore, previous research has demonstrated that measurement of cytokine levels in individuals with rheumatoid arthritis may be augmented by interfering factors such as rheumatoid factor [48, 49]; however, similar to previous cytokine research, not all adipokines may be influenced by such factors [50]. Rheumatoid factor was not measured in the current study, but further investigation into the potential interference of rheumatoid factor and other heterophilic antibodies with multiplex immunoassays should be considered for future work in this area.

## 5. Conclusions

In summary, the current nested case-control study examined the levels of adipokines in female participants with arthritis and obesity from a region of Canada that has particularly high levels of the two diseases. The findings demonstrated a significant correlation between various types of adiposity measures in participants with rheumatoid and osteoarthritis. Adipokines adiponectin, leptin, and resistin were analyzed in all groups; however, the only association was observed with adiponectin and those with osteoarthritis. Future research should focus on the complex relationship between adipokines and arthritis in obese individuals with specific types of arthritis, precisely how these levels change over time with disease progression. The Atlantic PATH cohort is intended to be longitudinal and data on participants will be collected for up to 30 years, opening the door for further exploration into the complex relationship between obesity and arthritis.

## Figures and Tables

**Table 1:** Characteristics of nested case-control participants with and without arthritis in the Atlantic PATH cohort.

	No arthritis	Rheumatoid	Osteoarthritis
	*n* (%)		
Age group			
50–59	49 (61.3)	35 (43.8)	36 (45.0)
60–69	31 (38.8)	45 (56.3)	44 (55.0)
Province			
Nova Scotia	43 (53.8)	40 (50.0)	38 (47.5)
New Brunswick	22 (27.5)	15 (18.8)	22 (27.5)
Newfoundland and Labrador	6 (7.5)	20 (25.0)	15 (18.8)
Prince Edward Island	9 (11.3)	5 (6.3)	5 (6.3)
Education			
High school or less	14 (17.5)	20 (25.3)	13 (16.3)
College level	24 (28.6)	33 (41.8)	27 (33.8)
University level	42 (52.5)	26 (32.9)	40 (50.0)
Smoking status			
Never	40 (50.0)	33 (41.3)	38 (47.5)
Former	35 (43.8)	42 (52.5)	35 (43.8)
Current	5 (6.3)	5 (6.3)	7 (8.8)
Alcohol drinking			
Abstainer	12 (15.0)	15 (18.8)	12 (15.0)
Occasional drinker	32 (40.0)	36 (45.0)	34 (42.5)
Regular drinking	23 (28.7)	24 (30.0)	18 (22.5)
Habitual drinker	13 (16.3)	5 (6.3)	16 (20.0)
Fruits & vegetables (five a day)	57 (71.3)	43 (53.8)	46 (57.5)
Diabetes	17 (3.8)	25 (8.8)	26 (7.6)
Hypertension	3 (21.3)	7 (32.1)	6 (32.5)
BMI > 30	40 (33.3)	40 (33.3)	40 (33.3)
Waist-to-hip ratio^a^	53 (66.3)	54 (67.5)	52 (65.8)
Abdominal obesity^b^	42 (52.5)	54 (67.5)	54 (68.4)
Physical activity level^c^			
Inactive	16 (20.0)	22 (27.8)	13 (16.3)
Moderately active	21 (26.3)	20 (25.3)	20 (25.0)
Active	43 (53.8)	37 (46.8)	47 (58.8)

	Mean (SD)		
Age	57.7 (5.1)	60.3 (5.6)^∗^	59.7 (5.1)^∗^
Duration of arthritis (years)	—	15.8 (15.7)^$^	10.5 (10.0)
Height, cm	166.0 (10.2)	166.5 (8.7)	165.1 (9.2)
Weight, kg	78.9 (24.2)	78.5 (20.3)	77.3 (20.1)
Waist, cm	87.3 (21.8)	92.8 (15.8)	92.4 (18.1)
Hips, cm	99.7 (21.8)	105.8 (14.7)	105.0 (16.2)
Body mass index (BMI), kg/m^2^	28.5 (7.6)	28.4 (7.6)	28.5 (7.7)
Waist-to-hip ratio	0.88 (0.1)	0.88 (0.1)	0.88 (0.1)
Percentage body fat, %	32.7 (9.9)	33.2 (11.2)	33.6 (10.5)
Fat mass index, kg/m^2^	9.8 (5.5)	9.9 (6.0)	10.2 (6.0)
BMI at 18 years	20.1 (4.3)	19.8 (3.4)	20.3 (4.1)
Change in BMI from 18 years	5.0 (6.5)	7.7 (6.2)	5.5 (6.1)

^a^Waist-to-hip ratio > 0.85 for women; ^b^waist circumference ≥ 88 cm for women; ^c^physical activity levels: inactive was classified as not meeting guidelines, and moderately active and active were classified as meeting guidelines of 150 minutes per week of moderate-to-vigorous physical activity. ^∗^Significant difference from no arthritis (*P* < 0.05) as determined by Kruskal-Wallis test; ^$^significant difference from osteoarthritis (*P* < 0.05) as determined by Student's *t*-test.

**Table 2:** Correlation between adiposity measures in nested case-control participants with and without arthritis in the Atlantic PATH cohort.

	Waist circumference	Body mass index (BMI)	Waist-to-hip ratio	Percentage body fat	Fat mass index	Change in BMI from 18 years
*No arthritis*						
Waist circumference	—	—	—	—	—	—
Body mass index (BMI)	0.185	—	—	—	—	—
Waist-to-hip ratio	**0.244** ^∗^	−0.079	—	—	—	—
Percentage body fat	0.164	**0.755** ^∗^	−0.065	—	—	—
Fat mass index	0.160	**0.930** ^∗^	−0.057	**0.903** ^∗^	—	—
Change in BMI from 18 years	0.145	**0.749** ^∗^	0.003	**0.323** ^∗^	**0.535** ^∗^	—
*Rheumatoid arthritis*						
Waist circumference	—	—	—	—	—	—
Body mass index (BMI)	−0.052	—	—	—	—	—
Waist-to-hip ratio	**0.577** ^∗^	−**0.224**^∗^	—	—	—	—
Percentage body fat	−0.049	**0.797** ^∗^	−**0.250**^∗^	—	—	—
Fat mass index	−0.101	**0.900** ^∗^	−**0.293**^∗^	**0.931** ^∗^	—	—
Change in BMI from 18 years	−0.136	**0.793** ^∗^	0.080	0.268	**0.481** ^∗^	—
*Osteoarthritis*						
Waist circumference	—	—	—	—	—	—
Body mass index (BMI)	0.179	—	—	—	—	—
Waist-to-hip ratio	**0.693** ^∗^	0.061	—	—	—	—
Percentage body fat	0.085	**0.807** ^∗^	−0.011	—	—	—
Fat mass index	0.148	**0.952** ^∗^	0.037	**0.935** ^∗^	—	—
Change in BMI from 18 years	0.128	**0.747** ^∗^	0.087	**0.365** ^∗^	**0.561** ^∗^	—

^∗^Significance at *P* < 0.001.

**Table 3:** Logistic regression models of adipokines as predictor of rheumatoid arthritis.

	Odds ratio (95% CIs)	*P* value
*Adiponectin*		
Unadjusted	1.059 (0.990–1.133)	0.096
Model 1	1.059 (0.989–1.135)	0.100
Model 2	1.048 (0.977–1.125)	0.188
*Leptin*		
Unadjusted	1.020 (0.994–1.047)	0.128
Model 1	1.016 (0.990–1.042)	0.222
Model 2	1.015 (0.989–1.041)	0.252
*Resistin*		
Unadjusted	1.041 (0.994–1.090)	0.092
Model 1	1.038 (0.990–1.087)	0.123
Model 2	1.042 (0.991–1.096)	0.109

Model 1: adjusted for age; model 2: adjusted for age, education, smoking, alcohol use, and fruit and vegetable intake.

**Table 4:** Logistic regression models of adipokines as predictor of osteoarthritis.

	Odds ratio (95% CIs)	*P* value
*Adiponectin*		
Unadjusted	1.071 (1.002–1.145)	0.042
Model 1	1.072 (1.002–1.147)	0.045
Model 2	1.072 (1.001–1.148)	0.045
*Leptin*		
Unadjusted	1.009 (0.994–1.037)	0.519
Model 1	1.005 (0.978–1.033)	0.709
Model 2	1.005 (0.978–1.028)	0.731
*Resistin*		
Unadjusted	1.021 (0.973–1.073)	0.396
Model 1	1.018 (0.970–1.070)	0.467
Model 2	1.022 (0.971–1.076)	0.402

Model 1: adjusted for age; model 2: adjusted for age, education, smoking, alcohol use, and fruit and vegetable intake.
